# Assessment of End User Computing Satisfaction (EUCS) of Electronic Hospital Management Information System (eHMIS) in Lady Reading Hospital Peshawar Pakistan

**DOI:** 10.12669/pjms.40.11.9156

**Published:** 2024-12

**Authors:** Faaiz Ali Shah

**Affiliations:** 1 Dr. Faaiz Ali Shah Associate Professor of Orthopaedics, Lady Reading Hospital, Peshawar, Pakistan

**Keywords:** Hospital Information System, Hospital Management Information System, User Satisfaction, End User Computing Satisfaction

## Abstract

**Objective:**

To determine user satisfaction of clinical faculty using Electronic Hospital Management Information System (eHMIS) in Lady Reading Hospital Peshawar Pakistan.

**Methods:**

This descriptive cross-sectional study was conducted in Orthopaedic Division Lady Reading Hospital Peshawar Pakistan from 15^th^ March 2023 to 15^th^ November 2023.All consultants serving in hospital fulfilling the inclusion criteria and consenting were included in this study. A validated questionnaire assessing the End User Computing Satisfaction (EUCS) was emailed to the included faculty. This questionnaire had 12 questions with answers ranked on the Likert scale (1 to 5 with 1 denoting almost never and five almost always). Data was analyzed with SPSS version 27.

**Results:**

We received 142 filled questionnaires. The mean age of our study participants was 43±2.1 years. Male participants were 128(90.14%) and female 14(9.85%). The eHMIS provided precise information to 112(78.87%) consultants most of the time. Majority (91.54%, n=130) of the respondents agreed that the information content met their need most of the time. Many (57.04%, n=81) consultants admitted that the output was presented in useful format most of the time and the information of was clear most of the time to 117(82.39%) consultants. Majority (69.71%, n=99) of the respondents got the information they needed in time almost always. The eHMIS provided up-to-date information almost always to 111(78.16%) respondents.

**Conclusion:**

Majority of our consultants were satisfied with the Electronic Hospital Management Information System (eHMIS) most of the time to almost always and most were using it several times daily.

## INTRODUCTION

Electronic Hospital Management Information System(eHMIS) is a computer based or online but paperless specialized, integrated and comprehensive information system for managing clinical, administrative and financial aspects of a hospital thus providing support for evidence based decisions in all spheres of patient health care service delivery.^1^ This system has many benefits. First, it can improve the quality of service delivery by achieving better clinical outcome with enhanced safety, efficacy and minimizing medical errors. Second, it saves resources and reduces treatment cost due to less manpower and paperless and filmless laboratory and radiology reports respectively. Third, it facilitates research and evidence-based decision because rapid and easy access to reliable and accurate data is ensured. Fourth, involvement of various specialties and stake holders by eliminating communication gap resulting in increased collaboration and coordination.^2^-^4^

In many developing countries including Pakistan attempts have been made to shift Hospital Management Information System from paper to paperless but the utilization and implementation of eHMIS is still low and limited and thus unable to provide the desired level of information for policy makers and health care providers.^5^,^6^ When measuring the success of eHMIS hospitals primarily focus on other aspects of information system and often neglect the human factors or forget to assess whether users are satisfied or pleased with it or not.^7^ Literature revealed that if users are satisfied with an information system they would continue to use that system positively. Both user satisfaction and continuous intention to use have positive impact on the user as well as on the organization. This is particularly important for developing countries like Pakistan where eHMIS is still in the initial phase of implementations and determining the behavior of health care providers would be more helpful in formulation of targeted interventional strategies.^8^

User Satisfaction (US) denotes user satisfactions with the web site, reports and support services of an information system and the extent to which the users are satisfied in getting the required information. The most widely used user satisfaction instrument is called the End User Computing Satisfaction(EUCS) proposed by Doll and Torkzadeh in 1988.^9^ The EUCS is an affective attitude and a useful tool to measure users experiences and expectations of an information system which they use directly and determined whether the information system was effective and fulfill users desires and expectations in reality or not.^10^ The EUCS is a standardized, validated and reliable instrument used globally to assess user satisfaction of an information system such as eHMIS.^11^ It is universally applicable to a variety of respondents, applications and all subset of population in any linguist and culture set up.^12^

We use eHMIS in our hospital which utilizes Oracle Products (Oracle Corporation, Redwoo) and was developed by Shaukat Khanum Memorial Cancer Hospital and Research Centre (SKMCH & RC) Lahore Pakistan over ten years period (2001 to 2011).^13^ The Board of Governors of Lady reading Hospital Peshawar had declared eHMIS use mandatory for all consultants of our hospital a year ago after the management had completed networking, equipment and training sessions to all the faculty.

Regular quarterly audit report of individual consultant usage of eHMIS are shared with faculty. Measuring user satisfaction of eHMIS in this involuntary situation seems more appropriate to determine success of implementation. We expect that an understanding of the EUCS of eHMIS the implementation will be made more easy, smooth and success ensured. To the best of our knowledge our study was the first study in Pakistan to determine the success of eHMIS using a standard and well known model of EUCS by Doll and Torkzadeh. Furthermore, our results of eHMIS evaluation would be used as a benchmark by the Government and policy makers to smoothly expand the implementation of eHMIS to others hospitals in our country.

## METHODS

This descriptive cross sectional study was conducted in Orthopaedic Division Lady Reading Hospital Peshawar Pakistan. The study extended from 15^th^ March 2023 to 15^th^ November 2023. Our sample size was 156 calculated with the help of Solvin’s formula^14^ [n=N/1+Ne^2^]^14^ whereas n=sample size, N=population size (219), e=margin of error (5%) and 10% non-respondent rate. Our sampling frame was all the consultants serving in our hospital and using eHMIS directly

### Ethical Approval:

The study protocol was approved by the Institutional Review Board (IRB) Lady Reading Hospital Peshawar (Ref. No. 681/LRH/MTI, dated March 9, 2023).

The data was obtained from Human Resource Department. Paramedics, nurses and managers were excluded. Incomplete questionnaire was also excluded. We adopted stratified probability sampling technique for data collection. The consultants were divided based upon their rank into Assistant Professors, Associate Professors, and Professors. Faculty in each rank was listed in alphabetical order and was assign a number. In each category simple random sampling technique was adopted through random number generator. The minimum proportion allocation in each category was 50% with minimum of one consultant per specialty at any rank. E-mails were send to the selected faculty explaining purpose of the eHMIS survey and asking their consent for participation in this survey by filling the attached questionnaire.

This questionnaire consisted of two parts in Table-I. Part one inquired about the demographic information of the respondents and frequency of usage of eHMIS while part two consisted of validated questionnaire proposed by Doll and Torkzadeh^9^ and has five first order variables namely Content(C), Accuracy(A), Format(F), Ease of Use(E) and Timeliness(T). These five robust variables were measured via 12 questions with answers ranked on the Likert scale (1-5 with one denoting almost never and five almost always). This 12-Item questionnaire had Reliability value of 0.92 and Criterion related Validity value of 0.76. This questionnaire had no Non-response Bias (earlier respondents versus delayed respondents versus non respondents) as shown by Independent Sample t-test result of 0.172 with significant p< 0.05.^9^,^15^

**Table-I:** Our study Questionnaire for assessing EUCS of eHMIS.

Name: Age:					
**Specialty: Designation:**					
**Year of passing postgraduate fellowship: Duration of service in this hospital:**					
Please click on the most appropriate **SINGLE** box(□)					
How frequently do you use eHMIS?					
□ A few times a month □					
□ A few times a week □					
□ A few times daily □					
□ Several times daily □					
□ Not at all □					
On average how much time do you spend using eHMIS?					
1 to 2 hours □					
3 to 4 hours □					
5 to 6 hours □					
>6 hours □					
**For each of the following questionnaire items click on the appropriate SINGLE box (□) that indicate your response.**					
*Questionnaire Items*	*Almost never*	*Some of the time*	*About half of the time*	*Most of the time*	*Almost always*
**Content(C)**					
C1: Does the system provide the precise information you need?	□	□	□	□	□
C2: Does the information content meet your needs?	□	□	□	□	□
C3: Does the system provide reports that seem to be just about exactly what you need?	□	□	□	□	□
C4: Does the system provide sufficient information?	□	□	□	□	□
**Accuracy(A)**					
A1: Is the system accurate?	□	□	□	□	□
A2: Are you satisfied with the accuracy of the system?	□	□	□	□	□
**Format(F)**					
F1: Do you think the output is presented in a useful format?	□	□	□	□	□
F2: Is the information clear?	□	□	□	□	□
**Ease of use(E)**					
El: Is the system user friendly?	□	□	□	□	□
E2: Is the system easy to use?	□	□	□	□	□
**Timeliness(T)**					
T1: Do you get the information you need in time?	□	□	□	□	□
T2: Does the system provide up-to-date information?	□	□	□	□	□

### Statistical Analysis:

We analyzed our data with SPSS version 27. Mean and standard deviation was calculated for quantitative variables like age and duration of service. Frequency and percentage was calculated for qualitative variables like gender and number of respondents and non-respondents. Comparative analysis of age, gender, rank, specialty, years of service, frequency and duration of eHMIS usage with questionnaire response rank was performed using Independent Sample t test and One way ANOVA.P value < 0.05 was considered significant. Data was presented in tables where necessary.

## RESULTS

In this study we e-mailed our study questionnaire to 156 consultants including Professors (n=7), Associate Professors (n=11) and Assistant Professors (n=138). We received 92 filled questionnaires after our first email and 55 after second reminder. The total positive respondents were 142(91.02%) while the non-respondents were 9(5.76%) and incomplete questionnaires were 5(3.20%). The non respondents and incomplete questionnaires were excluded from analysis.

Our final analysis included 142 questionnaires. The mean age of our study participants was 43±2.1 years. The mean duration of respondent’s service in this institution was 7.3±1.3 years. Male participants were 128(90.14%) and female 14(9.85%). Majority (62.67%, n=89) of the respondents were using eHMIS several times daily while 40(28.16%) were using a few times daily and 13(9.15%) a few times a week. About 95(66.90%) consultants were using eHMIS 1-2 hours and 47(33.09%) were using eHMIS 3-4 hours.

The frequency and duration of eHMIS usage was more in Assistant Professors than in Associate Professors and Professors. (p<0.05). Analysis of our questionnaire responses revealed that majority (72.53%, n=103) of our respondents opted for higher Likert scale answer options (3-5) of the 12 questions asked and thus indicated their favorable or positive End Using Computer Satisfaction (EUCS) of eHMIS used in our institution as shown in Table-II.

**Table-II:** Questionnaire response rate of our study participants.

S. No.	Questions	Almost never		Some of the time		About half of the time		Most of the time		Almost always	

		Number	Percentage	Number	Percentage	Number	Percentage	Number	Percentage	Number	Percentage
1.	C1	--	--	--	--	--	--	112	78.87%	30	21.12%
2.	C2	--	--	--	--	--	--	130	91.54%	12	8.45%
3.	C3	--	--	--	--	37	26.05%	45	31.69%	60	42.25%
4.	C4	--	--	--	--	19	13.38%	123	86.61%	--	--
5.	A1	--	--	--	--	123	86.6%	19	13.38%	--	--
6.	A2	--	--	11	7.74%	88	61.97%	43	30.28%	--	--
7.	F1	9	6.33%	--		52	36.61%	81	57.04%	--	--
8.	F2	--	--	9	6.33%	16	11.26%	117	82.39%	--	--
9.	E1	10	7.04%	--	--	132	92.95%	--	--	--	--
10.	E2	--	--	--	--	119	83.80%	--	--	23	16.19%
11.	T1	--	--	--	--	--	--	43	30.28%	99	69.71%
12.	T2	--	--	--	--	--	--	31	21.83%	111	78.16%

As per respondents reply the system provided precise information to 112(78.87%) consultants most of the time. Majority (91.54%, n=130) of the respondents agreed that the information content met their need most of the time. Some (42.25% n=60) consultants believed that the eHMIS provided reports that seemed to be just exactly what they needed most of the time. The system provided sufficient information about half of the time to 123(86.61%) consultants. The eHMIS system was accurate about half of the time in the opinion of 123(86.61%) consultants and 88(61.97%) consultants were satisfied with accuracy of the system most of the time.

Most (57.04%, n=81) consultants admitted that the output was presented in useful format most of the time but 52(36.61%) believed about half of the time and 9(6.33%) almost never. The information was clear most of the time to 117(82.39%) consultants. The system was user friendly and easy to use about half of the time to 132(92.95%) and 119(83.80%) consultants respectively. Majority (69.71%, n=99) of the respondents got the information they needed in time almost always. The eHMIS provided up-to-date information almost always to 111(78.16%) respondents and most of the time to 31(21.83%) respondents.

We had noted that favorable or higher Likert scale response rate had no statistically significant (p>0.05) association with respondents age, gender, rank, specialty, years of service, frequency and duration of eHMIS usage.

## DISCUSSION

The results of our study showed that vast majority of our respondents were satisfied with the content(C) of eHMIS most of the time and about half of the time with the accuracy(A) of eHMIS. The format(F) of eHMIS was satisfying to majority of our consultants most of the time. The ease of use(E) was satisfying to majority in about half of the time while the timeliness(T) of eHMIS was satisfying to majority of our respondents almost always. Literature revealed many studies from developing countries favoring eHMIS. Cucus and Halim^10^ conducted a study in Regional Public Hospital Dr. A. Dadi Tjokrodipo Bandar Lumpung, Indonesia involving 30 hospital faculty. Although these authors used the same questionnaire which we used in our study but they ranked the questionnaire responses into not very good (20% to 35%), not good (36% to 51%), fairly good (52% to 67%), good (68% to 83%), and very good (84% to 100%). These authors noted that all the variables namely Content, Accuracy, Format, Ease of Use and Timeliness are in good categories with mean of 55.67%, 60.27%, 62.50%, 64.83% and 66.17% respectively. They concluded that overall user satisfaction of eHMIS was in good category with mean value of 71.33%. Karitis et al.^16^ assessed the eHMIS of General Hospital of Chios Greece. The number of survey respondents were 71. The questionnaire was based on DeLone and McLean (D & M) Model which consider EUCS as an important indicator of eHMIS success determination. Karitis et al. concluded that the information system was successful as users were satisfied. This study also postulated that the more the users are satisfied with the system the more they will continue to use it. Antissha et al.^17^ determined the factors affecting the success of implementation of eHMIs in Arsani Hospital Indonesia using Human Organizational and Technology Fit Model (HOT-FIT). This hospital had implemented eHMIS since 2010. Based upon the feedback questionnaire of 199 health care workers of the hospital they concluded that the net benefits of eHMIS usage was affected by user satisfaction. If users were satisfied with eHMISS they would feel its benefits and would continue to use it.

Another study conducted in Tanzania explored the predictors of success of eHMIS after taking questionnaire feedback from their 69 hospitals staff (both clinical and non-clinical). This study reported that majority (81.2%) of the hospital staff were using eHMIS and that user satisfaction had a positive influence on the intention to continued usage of eHMIS.^18^ One study based upon the feedback from 228 end users belonging to 11 hospitals in Iran revealed that the user’s satisfaction level was good with mean satisfaction score was 54.6%.^19^

In our study few consultants (6.33%, n=9) were of the opinion that eHMIS output was almost never presented in a useful format while only 10(7.04%) consultants pointed out that system was almost never user friendly to them. Few other studies had reported similar results. Sanjuluca et al.^20^ conducted a cross sectional study in seven hospitals of Angola and gathered data from 36 hospital employees. The author found that two thirds of the respondents were not satisfied with eHMIS. Another Iranian study^21^ surveyed 155 staff of Doctor Gholipur Hospital and 80 staff of Emam Ali hospital about users’ satisfaction of eHMIS with regard to software quality, quality of information, data and functions of the system. This study found that user satisfaction was average in all dimensions measured. The most probable reason for this low user satisfaction in this study was that both the hospitals were using eHMIs and paper based conventional system side by side.

Herwati et al.^22^ surveyed 35 health care professionals regarding EUCS of eHMIS and documented that majority (43%, n=15) of the participants were very satisfied with eHMIS,13(37%) was satisfied and 7(20%) were quite satisfied with eHMIS. Amongst the five components of EUCS, content (p=0.001) and timeliness(p=0.003) statistically influenced user satisfaction than other components of EUCS. Majority (67%, n=24) of the respondents in this study however were females. Meiyana et al. and colleagues^23^ determined EUCS of eHMIS of 105 healthcare professionals and reported a significant association between user satisfaction and all the five components of EUCS(p=0.001). Those healthcare professionals who attended computer courses(p=0.022) and female professionals(p=0.007) were more likely to positively report ease of use content than other professionals. These authors concluded that healthcare professionals should have computer training so that ease of use and EUCS of eHMIS is ensured in hospitals.

In Pakistan currently eHMIS has been in regular use in very few well reputed tertiary care public and private sector hospitals like Shaukat Khanum Memorial Cancer Hospital and Research Centre (SKMCH & RC) Lahore, Sindh Institute of Urology and Transplantation (SUIT), Gurki Trust Teaching Hospital Lahore (GTTH) and Indus Hospital Karachi.^24^ The SKMCH & RC published the development and financial benefits of eHMIS. 13

### Strength and Limitations of the study:

The strength of our study was the large sample size consisted of consultants of variable experience, ranks and diverse specialties and the utilization of a standard validated and well known EUCS questionnaire for data collection. To the best of our knowledge this was the first study in Pakistan using EUCS for determining success of eHMIS. This study was conducted in the largest public sector hospital of our province where eHMIS usage was mandatory for all consultants.

### Limitations of the study:

First our study did not involve other healthcare providers like nurses, paramedics and managers who are also actively and routinely using eHMIS in our institution. Second, employee’s individual motivations and commitment to use eHMIS was not assessed in our study. Third, we used only questionnaire to determine the success of implementation of eHMIS in our study but it would be more appropriate if personal interviews, focus group discussion and questionnaire were used in combination for assessment of eHMIS. Fourth, we were not able to determine the computer literacy skills of our respondents because individuals with higher computer literacy skills might be more satisfied with eHMS than with low computer literacy. Further studies are therefore recommended.

## CONCLUSION

Majority of our consultants were satisfied with the Electronic Hospital Management Information System(eHMIS) most of the time to almost always and most were using it several times daily. This application had fulfilled user satisfaction by fulfilling their desired requirements and can be used in other tertiary care public hospitals with full confidence and success of implementation ensured.

## References

[1] Gesulga JM, Berjame A, Moquiala KS, Galido A (2017). Barriers to Electronic Health Record System Implementation and Information Systems Resources:A Structured Review. Procedia Comput Sci.

[2] AbouZahr C, Boerma T (2005). Health information systems:the foundations of public health. Bull World Health Organ.

[3] Aiga H, Kuroiwa C, Takizawa I, Yamagata R (2008). The reality of health information systems:challenges for standardization. Biosci Trends.

[4] Pare G, Sicotte C (2001). Information technology sophistication in health care:an instrument validation study among Canadian hospitals. Int J Med Inform.

[5] Gebre-Mariam M, Bygstad B (2019). Digitalization mechanisms of health management information systems in developing countries. Inform Organ.

[6] Upadhaya N, Jordans MJD, Abdulmalik J, Ahuja S, Alem A, Hanlon C (2016). Information systems for mental health in six low and middle income countries:Cross country situation analysis. Int J Ment Health Syst.

[7] DeLone WH, McLean ER (2016). Information Systems Success Measurement. Found Trends Inform Syst.

[8] Lwoga ET, Sangeda RZ, Mushi R (2020). Predictors of electronic health management information system for improving the quality of care for women and people with disabilities. Inform Develop.

[9] Doll WJ, Torkzadeh G (1988). The measurement of end user computing satisfaction. MIS Quarterly.

[10] Cucus A, Halim G (2019). Testing User Satisfaction Using End-User Computing Satisfaction (EUCS) Method in Hospital Management Information System (SIMRS) (Case Study at the Regional Public Hospital. J Inform Eng Appl.

[11] Doll WJ, Torkzadeh G (1991). The measurement of end-user computing satisfaction:theoretical and methodological issues. MIS Quarterly.

[12] Aggelidis VP, Chatzoglou PD (2012). Hospital information systems:measuring end user computing satisfaction (EUCS). J Biomed Inform.

[13] Sultan F, Aziz MT, Khokhar I, Qadri H, Abbas M, Mukhtar A (2014). Development of an in-house hospital information system in a hospital in Pakistan. Int J Med Inform.

[14] Tejada JJ, Punzalan JRB (2012). On the Misuse of Slovin's Formula. Philipp Stat.

[15] Doll WJ, Deng X, Raghunathan TS, Torkzadeh G, Xia W (2004). The Meaning and Measurement of User Satisfaction:A Multigroup Invariance Analysis of the End-User Computing Satisfaction Instrument. J Manag Inform Sys.

[16] Karitis K, Gallos P, Triantafyllou IS, Plagianakos V (2021). Chios Hospital Information System Assessment. Stud Health Technol Inform.

[17] Antissha D, Azizah AH, Arifin S (2022). Assessing Hospital Management Information Systems Success Using Human Organization and Technology Fit Model. Appl Inf Syst Manag (AISM).

[18] Lwoga ET, Sangeda RZ, Mushi R (2020). Predictors of electronic health management information system for improving the quality of care for women and people with disabilities. Inf Develop.

[19] Saghaeiannejad-Isfahani S, Jahanbakhsh M, Habibi M, Mirzaeian R, Nasirian M, Rad JS (2014). A Survey on the Users'Satisfaction with the Hospital Information Systems (HISs) based on DeLone and McLean's Model in the Medical-Teaching Hospitals in Isfahan City. Acta Inform Med.

[20] Sanjuluca THP, de Almeida AA, Cruz-Correia R (2022). Assessing the Use of Hospital Information Systems (HIS) to Support Decision-Making:A Cross-Sectional Study in Public Hospitals in the Huíla Health Region of Southern Angola. Healthcare.

[21] Fathi F, Dezhman S (2017). Study of Level of users'Satisfaction with Hospital Information System in Doctor Gholipur and Emam Ali Hospital in Boukan city in 2016-2017. Spec J Med Res Health Sci.

[22] Herwati I, Ayu JP, Mustafida L (2023). User Satisfaction Analysis of Hospital Management Information System Using the EUCS Method at Mitra Delima Hospital. Jurnal Manajemen Kesehatan Indones.

[23] Meiyana NS, Susanto T, Rokhmah D, Yunanto RA, Rahmawati I (2023). Analysis of hospital management information systems satisfaction using the end-user computing satisfaction method:A cross-sectional study. Jurnal Keperawatan Padjadjaran.

[24] Junejo S, Shahid S, Khursheed N, Maqsood S, Adnan F, Khalid F (2023). Cholera outbreak in 2022 among children in Karachi:Study of cases attending to a Tertiary Care Hospital. Pak J Med Sci.

